# A comparison of dimensional and discrete models for the representation of perceived and induced affect in response to short musical sounds

**DOI:** 10.3389/fpsyg.2023.1287334

**Published:** 2023-10-31

**Authors:** Iza Ray Korsmit, Marcel Montrey, Alix Yok Tin Wong-Min, Stephen McAdams

**Affiliations:** ^1^Music Research Department, Schulich School of Music, McGill University, Montreal, QC, Canada; ^2^Department of Psychology, McGill University, Montreal, QC, Canada

**Keywords:** music, emotion, affect, dimensional, discrete, categorical, individual differences

## Abstract

**Introduction:**

In musical affect research, there is considerable discussion on the best method to represent affective response. This discussion mainly revolves around the dimensional (valence, tension arousal, energy arousal) and discrete (anger, fear, sadness, happiness, tenderness) models of affect. Here, we compared these models' ability to capture self-reported affect in response to short, affectively ambiguous sounds.

**Methods:**

In two online experiments (*n*_1_ = 263, *n*_2_ = 152), participants rated perceived and induced affect in response to single notes (Exp 1) and chromatic scales (Exp 2), which varied across instrument family and pitch register. Additionally, participants completed questionnaires measuring pre-existing mood, trait empathy, Big-Five personality, musical sophistication, and musical preferences.

**Results:**

Rater consistency and agreement were high across all affect scales. Correlation and principal component analyses showed that two dimensions or two affect categories captured most of the variation in affective response. Canonical correlation and regression analyses also showed that energy arousal varied in a manner that was not captured by discrete affect ratings. Furthermore, all sources of individual differences were moderately correlated with all affect scales, particularly pre-existing mood and dimensional affect.

**Discussion:**

We conclude that when it comes to single notes and chromatic scales, the dimensions of valence and energy arousal best capture the perceived and induced affective response to affectively ambiguous sounds, although the role of individual differences should also be considered.

## 1. Introduction

One of the main reasons why people listen to music is its ability to convey and induce moods and emotions (Juslin and Laukka, 2004). In musical emotion research, however, the theoretical representation and practical operationalization of emotion is a constant source of discussion and discrepancy in findings. This discussion mirrors the discourse in general emotion research and mostly revolves around the distinction between discrete (or categorical) basic emotions (Ekman, 1999) vs. multidimensional affect (Russell, 1980). These differences in theoretical and practical approaches complicate comparisons between findings and inhibit discourse about the musical features and mechanisms that underlie affective response to music. Affect, here, is used as an umbrella term that covers both (subtle, long-term) moods and (intense, short-term) emotions, as well as other valenced states (Juslin and Västfjäll, 2008). This paper compares the suitability of the discrete and dimensional emotional models for representing perceived and induced musical affect.

Theories of basic emotions as discrete categories in their strictest form suggest that there are several basic emotions that are both universal and innate, such as anger, fear, sadness, happiness, and disgust. Each of these emotions is supported by independent neural systems and expressed categorically (Ekman, 1999; Panksepp, 2007). Research shows that certain basic emotions are readily perceived and induced when listening to music (Juslin and Laukka, 2003, 2004; Juslin and Timmers, 2010). However, the evidence to support the basic emotion hypothesis, that independent neural systems underlie discrete emotions, has been unreliable and inconsistent (Cacioppo et al., 2000; Barrett, 2006). Furthermore, certain basic emotions are less commonly perceived or induced in a musical context, such as disgust. This category is often replaced by more musically applicable emotions such as peacefulness or tenderness (Gabrielsson and Lindström, 1995; Vieillard et al., 2008; Juslin and Timmers, 2010). The Geneva Emotional Music Scale (GEMS) is a model of discrete affect specifically tailored to the musical domain, with a nine-factor model consisting of wonder, transcendence, power, tenderness, nostalgia, peacefulness, joyful activation, sadness, and tension (Zentner et al., 2008).

Studies on musical emotions have increasingly employed a version of the dimensional model of affect (Eerola and Vuoskoski, 2013). The most common dimensional affect representation is that of valence (displeasure/pleasure) and arousal (deactivation/activation) in the circumplex model (Russell, 1980). Some studies operationalize valence as a negative/positive bipolar dimension (e.g., Vuoskoski and Eerola, 2011a), although this is found to be strongly correlated with the displeasure/pleasure operationalization (McAdams et al., 2017). Like discrete emotions, affects that occupy one of the four quadrants of the two-dimensional affect model (positive/negative valence and high/low arousal) are readily recognized in and induced by music (Rickard, 2004; Ilie and Thompson, 2006; Vieillard et al., 2008; Eerola et al., 2012; McAdams et al., 2017). One criticism of the two-dimensional affect model is that it cannot distinguish between certain emotions. For example, although anger and fear are distinct emotions, they would theoretically be positioned on the same valence and arousal coordinates. Thus, tension arousal has been employed as a third dimension of the affect space in music research. However, although some studies find that tension arousal is a useful addition (Ilie and Thompson, 2006; McAdams et al., 2017), others find that it does not improve the performance of two-dimensional models of perceived and induced affect (Eerola and Vuoskoski, 2011; Vuoskoski and Eerola, 2011a; Eerola et al., 2012).

Hybrid models combine the discrete emotion and dimensional affect models by suggesting that fundamentally, core affect is dimensional in nature, but the conscious interpretation or appraisal of these affects may be described in categorical terms (Russell, 2003; Barrett, 2006). Thus, the suitability of the dimensional or discrete representation of affect would depend on whether one is investigating core affect or emotional episodes. Moreover, individual differences exist in how people label affective states, some fitting better into a dimensional and others into a discrete representation (Barrett, 1998).

Stimulus selection is important when comparing the suitability of different affect models because they determine the kind of affective variation that can be observed or felt in response to stimuli. Eerola and Vuoskoski (2011) created a stimulus set that consisted of examples that were representative of discrete emotions, as well as examples that were representative of the extremes of valence, energy arousal, and tension arousal, as confirmed by listener judgments. They found that for perceived affect, two dimensions of affect (labeled valence and arousal) provided the highest correspondence between the dimensional and discrete affect ratings, and that the discrete emotion model performed especially poorly in characterizing the affective content of emotionally ambiguous examples. Using stimuli from the same corpus, they also compared the discrete, dimensional, and GEMS models on induced musical affect (Vuoskoski and Eerola, 2011a). Here, too, they found that the two-dimensional model of affect outperformed the other two models in terms of rating consistency and discrimination of music excerpts. Although Vuoskoski and Eerola assembled a corpus that spans both discrete and dimensional continua, it is possible that these selections lacked variation on some dimensions or categories that were not pre-determined by the authors.

The current study uses a selection of relatively short sounds that were not selected based on their affective intent, but rather on their variation in timbre, pitch, and stimulus length, as is typical of stimuli used in affect research to investigate local musical features. Such sounds may be considered more affectively ambiguous, as they are not played to explicitly communicate specific affective content. This study aims to investigate the applicability of the discrete and dimensional affect models to musical sounds that are not selected or designed with a specific affective intent. Applicability of the affect models can be assessed in terms of participants' preference (e.g., Zentner et al., 2008) or cross-cultural consistency (e.g., Cowen et al., 2020). However, here we focus on an English-speaking population and take an approach similar to previous studies (Eerola and Vuoskoski, 2011; Vuoskoski and Eerola, 2011a) by assessing applicability in terms of the following criteria: (1) consistency and agreement among raters, (2) model redundancy, (3) correspondence between the two models (mapping), and (4) the influence of individual differences.

We test these criteria on two different stimulus sets (single notes and chromatic scales) and on both affect loci (perceived and induced), comparing the dimensional and discrete affect model. The dimensional model comprises all three dimensions of valence, tension arousal, and energy arousal. Different variations of the discrete model have been used in previous research (Gabrielsson and Lindström, 1995; Vieillard et al., 2008; Zentner et al., 2008; Juslin and Timmers, 2010). We expect that the GEMS-model will show high redundancy in response to the relatively short musical sounds we used in this study. Therefore, we follow the use of the five discrete categories of anger, fear, happiness, tenderness, and sadness to be comparable to previous studies (Eerola and Vuoskoski, 2011; Vuoskoski and Eerola, 2011a).

We hypothesize that the dimensional model is more effective at capturing the affectively ambiguous short sounds, but that an increased stimulus length from single notes to chromatic scales would increase the likelihood of affective appraisal, making the discrete model more effective at capturing the affective response. Furthermore, the perceived and induced affective responses to music are not necessarily the same, and they may differ both in strength and direction (Gabrielsson, 2001). Thus, here we may see differences in terms of applicability as well. We hypothesize that the perception of emotions may warrant more discrete categorization than induced feelings of smaller changes in mood, which may be represented as dimensional core affect. Finally, we will explore the role of individual differences—personality, mood, and musical preferences—in the affective response to music.

## 2. Methods

In two online experiments, we systematically varied stimulus length from single notes (Exp 1) to 15-note chromatic scales (Exp 2). Different groups of participants rated affect on three dimensional scales (valence, tension arousal, and energy arousal) or five discrete scales (anger, fear, sadness, happiness, and tenderness). Separate groups rated perceived and induced affect in Exp 1, whereas the same group rated perceived and induced affect in separate blocks in Exp 2.

### 2.1. Inclusion and exclusion

Participants were recruited through Prolific (www.prolific.co). Prolific's inclusion criteria determined that participants spoke fluent English and had no hearing problems. Before starting the experiment, participants were asked to only participate if they would use earphones or headphones and run the experiment on a laptop or desktop computer. Participants were excluded from the analysis if one or both of the following criteria were fulfilled: (1) their instruction reading times were unrealistically fast; (2) their rating data showed a combination of fast trial reaction time and high uniformity across stimuli and scales. To explain the second criterion, if a participant completed their ratings very quickly and a given stimulus was rated similarly on all five discrete affect scales (i.e., equally angry, fearful, sad, happy, and tender), it is likely that they were just “clicking through” the experiment and did not provide trustworthy data. Reaction times of one standard deviation below average were deemed too fast. High uniformity, i.e., whether participants appeared to always give the same response on each affect scale, was determined by low variability on affect scales across stimuli (low being one standard deviation below the group average variability).

### 2.2. Participants

Following the exclusion of 33 participants (11%) in Exp 1, we obtained rating data from 263 participants randomly assigned to rate perceived dimensional (*n* = 67), perceived discrete (*n* = 65), induced dimensional (*n* = 65), or induced discrete affect (*n* = 66). As gender identity, 161 indicated male (61%), 96 female (37%), 4 non-conforming or questioning (2%), and 2 preferred not to answer (<1%). Ages varied in a wide range from 18 to 68 years old (*M* = 29.0, *SD* = 10.2). Most participants grew up in Europe (71%), followed by North America (17%), Africa (5%), South America (3%), Asia (3%), and Oceania (<1%). The completed educational level of most participants was a bachelor's degree (33%), followed by high-school (31%), Master's (19%), secondary school (7%), T-levels (UK-based; 7%), PhD (2%), and other/NA (1%).

Following the exclusion of 29 participants (16%) in Exp 2, we obtained rating data from 152 participants randomly assigned to rate dimensional (*n* = 76) or discrete (*n* = 76) affect (note that all participants rated both affect loci here). As gender identity, 84 indicated male (55%), 67 female (44%), and 1 preferred not to answer (<1%). Again, ages varied widely from 18 to 68 years old (*M* = 31.6, *SD* = 10.4). Most participants spent their formative years in Europe (48%), followed by North America (36%), Africa (14%), Asia (2%), and South America (<1%). Most participants completed a bachelor's degree (36%), followed by Master's (25%), high school (18%), T-levels (UK-based; 9%), secondary school (8%), PhD (2%), and other/NA (3%).

### 2.3. Materials

#### 2.3.1. Stimuli

For Exp 1, stimuli were obtained from the Vienna Symphonic Library (VSL; Vienna Symphonic Library GmbH, 2022) and consisted of 59 recordings of a single note played at pitch class D# at a *forte* dynamic, varying in orchestral instrument family (woodwind, brass, strings, pitched percussion) and pitch register (1–7, where C4 is middle C with a fundamental frequency of 261.6 Hz), for a duration of 3 s. For Exp 2, we used OrchSim (OrchPlayMusic, 2022) to create 32 stimuli that played a 15-note chromatic scale (ascending and descending) spanning a perfect fifth (C–G–C), tenuto style at a *mezzo-forte* dynamic with added reverb of a medium-sized room and a duration of ~8 s. Stimuli varied in the same orchestral instrument families as in Exp 1 and in pitch register (1–6, to accommodate the perfect fifth range).

#### 2.3.2. Affect ratings

The dimensional affect group rated each stimulus on valence (negative/positive), tension arousal (relaxed/tense), energy arousal (tired/awake), as well as preference (dislike/like) on 9-point analogical-categorical scales (Weber, 1991), with *positive, relaxed, awake*, and *like* on the right-hand side of the scale. The discrete affect group rated each stimulus in terms of anger, fear, sadness, happiness, and tenderness on scales ranging from “no [anger/fear/sadness/happiness/tenderness]” to “a lot of [anger/fear/sadness/happiness/tenderness]”. They did not rate preference, because rating five scales can already be considered a high cognitive load. In the perceived condition, all participants were asked “What emotional quality do you perceive in this sound?” and in the induced condition, they were asked, “What emotional quality do you feel in response to this sound?”.

#### 2.3.3. Questionnaires

Measures of individual differences were collected through questionnaires on pre-existing mood (PANAS-X; Watson et al., 1988), Big-Five personality (BFI-44; John and Srivastava, 1999), trait empathy (IRI; Davis, 1983), musical sophistication (Gold-MSI; Müllensiefen et al., 2014), and music preferences (STOMP-R; Rentfrow et al., 2011).

### 2.4. Procedure

Prolific participants were redirected to an external link hosted on a secure webserver at McGill University. The experimental interface was built with JavaScript. Before starting the experiment, participants gave their informed consent in an online form. A headphone test was conducted to ensure that participants were able to hear sound in both ears. Based on a few test sounds, participants were then asked to set their volume to a level that ensured the sounds were audible but not uncomfortably loud. The instructions explained the difference between perceived and induced affect and which locus the participants would be rating during the listening task. Participants then completed the PANAS-X to measure pre-existing mood. Next, they completed the listening task. The order in which the rating scales were presented on screen was randomized for each participant but remained consistent throughout the experiment. The preference scale was randomly presented either above or below the three dimensional affect scales. Stimuli were also presented in random order. In Exp 2, the order of the perceived and induced blocks was randomized, and the dimensional group only rated preference in the perceived affect block. After completing the listening task, participants answered demographic questions and filled out the remaining questionnaires. Participants were financially compensated, and the protocol was certified for ethical compliance by the McGill University Research Ethics Board II. All data analyses were done in R version 4.2.1 (www.r-project.org).

## 3. Results

### 3.1. Consistency and agreement

**Figure 1:**
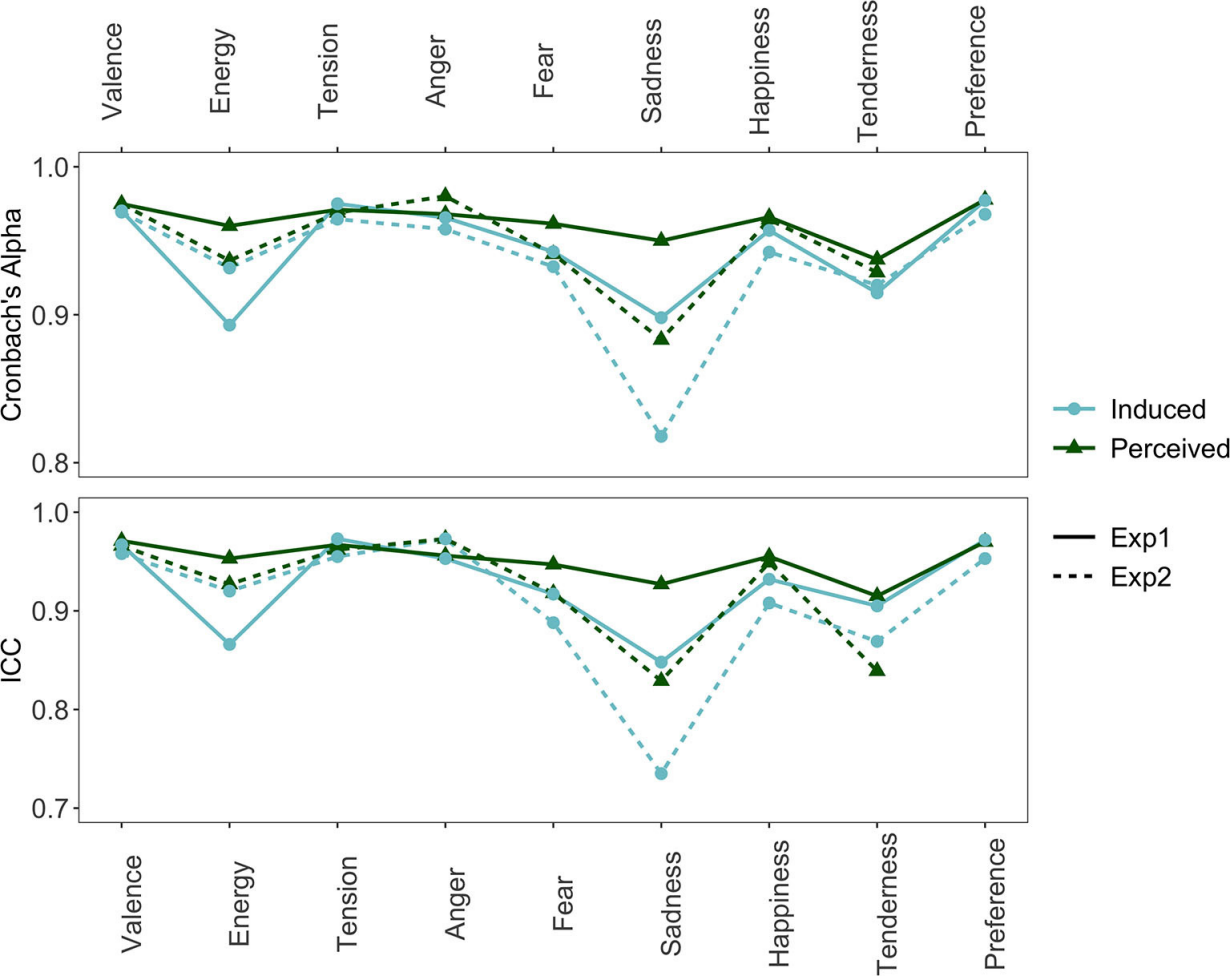
Cronbach's alpha and intraclass correlation (ICC) of each affect scale separated by affect locus and experiment.

Figure 1 shows the internal consistency (Cronbach's alpha) and the inter-rater agreement (intraclass correlation; ICC) for each of the affect scales. Whereas, Cronbach's alpha measures consistency, regardless of individual participants' mean ratings (some participants tend to give higher scores than others), ICC measures both the correlation between participants' ratings and how similar their mean ratings are. Both Cronbach's alpha and ICC show highly similar results with good consistency and agreement [range alpha = (0.82, 0.98); range ICC = (0.74, 0.97)]. Thus, participants' ratings across stimuli were both strongly correlated and highly similar. We can see, however, that averaged over affect loci and experiments, sadness scores the lowest on both Cronbach's alpha (*M* = 0.89; *SD* = 0.05) and ICC (*M* = 0.83, *SD* = 0.08), with the largest dip in internal consistency for induced sadness in Experiment 2 (alpha = 0.82, ICC = 0.74). Consequently, sadness, may be the most susceptible to individual differences. Preference shows the highest consistency and agreement overall (*M*_*alpha&ICC*_= 0.97, *SD*_*alpha&ICC*_= 0.01), followed by valence and tension arousal. Averaged over affect locus, experiment, and affect model (excluding preference), consistency and agreement were slightly higher for perceived compared to induced affect, for Exp 1 compared to Exp 2, and for the dimensional compared to the discrete affect model, but these differences were very minor and possibly negligible [Δ *alpha/ICC* = (0.01,0.03)]. We nevertheless report them here because previous studies (e.g., Eerola and Vuoskoski, 2011) also reported similarly small differences in the same direction (see Section 4).

### 3.2. Model redundancy

Table 1 shows the Pearson correlations between scales within each of the dimensional and discrete affect models, separated by experiment and affect locus. For each pair of correlations, we tested whether there was a difference in magnitude of affect loci between experiments. The comparison of affect loci entailed testing the difference between two non-overlapping dependent groups (different variables on the same stimuli). The comparison of experiments entailed testing the difference between independent groups (different variables and different stimuli for the same instrument). For both kinds of comparisons, several different tests are possible, as discussed by Diedenhofen and Musch (2015). Here, all tests suggested by Diedenhofen and Musch agreed on the statistical (non)significance of the difference between correlations.

**Table 1:** Pearson correlations between scales within dimensional and discrete affect models per affect locus and experiment.

**Scale pairs**	**Experiment 1 (*****df*** = **57)**		**Experiment 2 (*****df*** = **30)**	
	**Perceived**	**Induced**	**Perceived**	**Induced**
Valence–tension	−0.89^***^	−0.96^***^	−0.95^***^	−0.97^***^
Valence–energy	0.22	−0.23	0.28	0.18
Valence–preference	0.88^***^	0.97^***^	n/a	0.94^***^
Tension–energy	0.22	0.45^**^	−0.02	0.04
Tension–preference	−0.94^***^	−0.98^***^	n/a	−0.94^***^
Energy–preference	−0.14	−0.38^**^	n/a	−0.00
Anger–fear	0.90^***^	0.70^***^	0.87^***^	0.90^***^
Anger–sadness	0.47^**^	0.56^***^	0.48^*^	0.45^*^
Anger–happiness	−0.85^***^	−0.80^***^	−0.82^***^	−0.81^***^
Anger–tenderness	−0.90^***^	−0.76^***^	−0.91^***^	−0.90^***^
Fear–sadness	0.51^***^	0.59^***^	0.49^*^	0.38^*^
Fear–happiness	−0.84^***^	−0.74^***^	−0.88^***^	−0.76^***^
Fear–tenderness	−0.84^***^	−0.79^***^	−0.89^***^	−0.81^***^
Sadness–happiness	−0.80^***^	−0.82^***^	−0.77^***^	−0.76^***^
Sadness–tenderness	−0.56^***^	−0.63^***^	−0.62^**^	−0.54^*^
Happiness–tenderness	0.90^***^	0.92^***^	0.93^***^	0.92^***^

* *p* < 0.05;

** *p* < 0.001;

*** *p* < 0.0001.

The only consistent pattern of differences between correlations is found in Experiment 1, where all correlations within the dimensional affect scales were significantly stronger for induced affect than perceived affect (all *p* < 0.001, average Δ|*r*| = 0.09). This was not the case for any of the dimensional affect scales in Experiment 2 (average Δ|*r*| = 0.04). For the discrete affect scales, correlations in the perceived and induced loci were rarely significantly different from each other (average Δ|*r*| = 0.07). When comparing the correlations between experiments, there were also only rarely significant differences between the correlations (average Δ|*r*| = 0.07). Because the correlations in both experiments and affect loci are so close to each other and the statistical differences we do find concern differences in strength and not direction, we can expect that further analysis of model redundancy will lead to similar results as well. Thus, for further comparisons of the affect models, we combined the data of the two affect loci and experiments, although the differences between affect loci in the first experiment are still considered.

**Figure 2:**
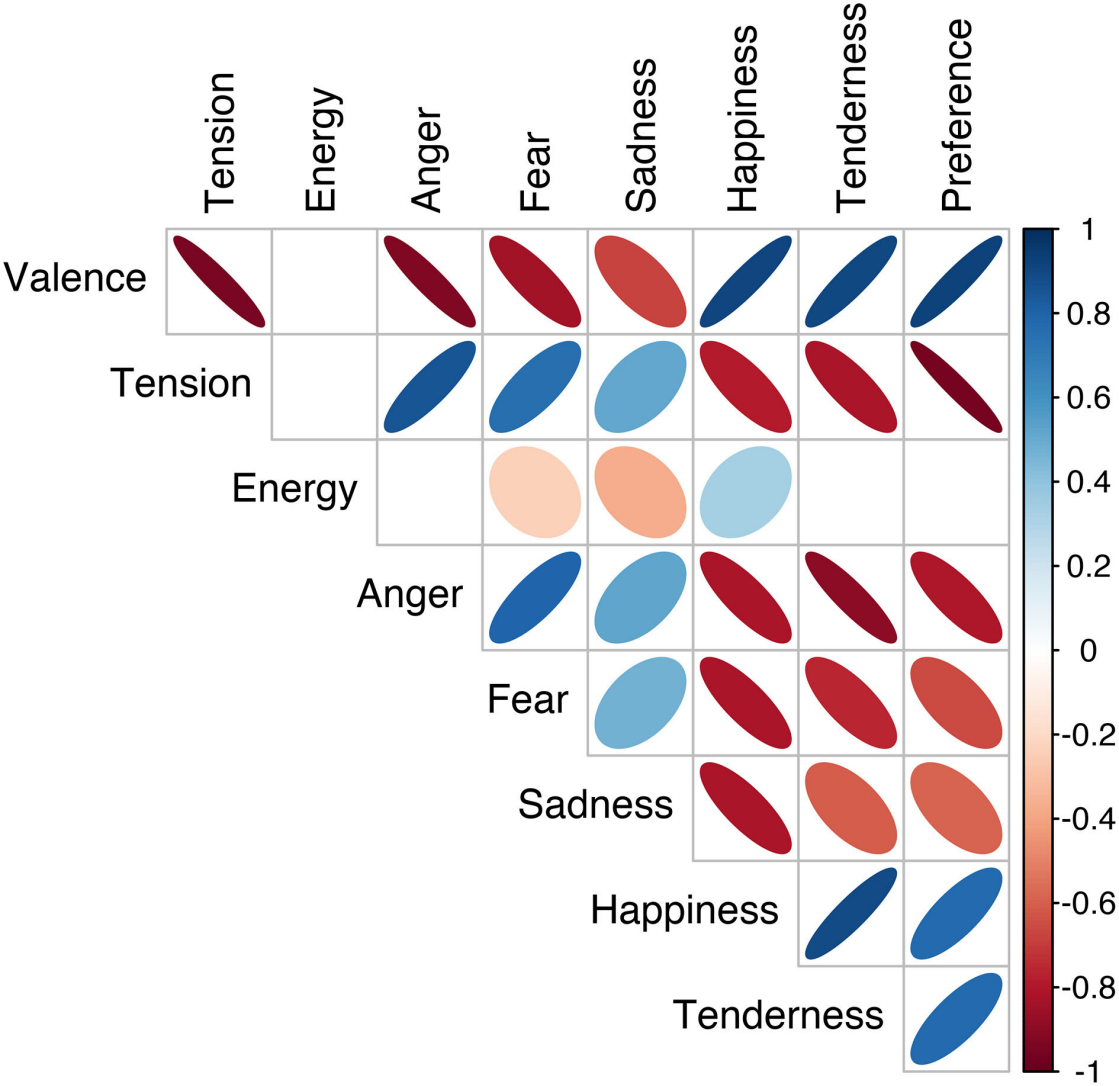
Pearson correlations between pairs of affect scales: orientation and color (red/blue) of ellipses indicate correlation direction, and narrowness and color saturation indicate correlation strength. Ellipses are only visualized for statistically significant correlations (*p* < 0.05).

Figure 2 visualizes the correlation of all the affect scales, including correlations between affect scales from the dimensional and discrete models, averaged over experiment and affect locus. All significant correlations (*p* < 0.05) are represented by an ellipse. The color (blue/red) and the orientation (increasing/decreasing) of the ellipse signify the correlation direction (positive/negative, respectively), and the narrowness and color saturation signify the strength of correlation (i.e., narrower and more saturated for stronger correlations). Nearly all scales were significantly correlated with each other. Within the dimensional affect scales, there is a strong negative correlation between valence and tension arousal, *r*(89) = −0.94, *p* < 0.0001, but no significant correlation with energy arousal. Within the discrete affect scales, anger, fear, happiness, and tenderness were relatively strongly correlated with each other, *|r|*(89) = (0.75, 0.90), *p* < 0.0001. Although sadness was also strongly correlated with happiness, *r*(89) = −0.81, *p* < 0.0001, it was less strongly correlated with the other discrete affect scales, *|r|*(89) = (0.48, 0.60), *p* < 0.0001. This is our first indication that in both models, some of the three dimensions and five categories may be redundant.

There were also strong correlations between the dimensional and discrete affect models. Valence and tension were strongly correlated with anger, fear, happiness, and tenderness, *|r|*(89) = (0.76, 0.92), *p* < 0.0001, but less strongly with sadness, *|r|*(89) = (0.51, 0.68), *p* < 0.0001. Energy did show correlations with fear, sadness, and happiness, but these were relatively weak, *|r|*(89) = (0.24, 0.36), *p* < 0.01. Preference was also correlated with most affect scales, except for energy arousal. It was most strongly correlated with tension arousal, *r*(89) = −0.95, *p* < 0.0001, and less strongly correlated with sadness, *r*(89) = −0.59, *p* < 0.0001. To summarize, nearly all affect scales were strongly correlated with each other, except for energy arousal and sadness, which showed non-significant or weaker correlations.

We used principal component analysis (PCA) to investigate how the three dimensional and five discrete affect scales could be reduced to a lower number of principal components (PCs). We did this for the dimensional and discrete affect scales individually, as well as combined (hybrid). Table 2 shows the results of the three PCAs. In all three cases, two PCs explained most of the variance in the rating data. For the dimensional model, the loadings of the three dimensional scales indicate that the first component is represented by valence and tension arousal in opposite directions, whereas the second component is represented by energy arousal. We also considered the PCA for the perceived and induced dimensional model in the first experiment separately (not described in Table 2), because of our earlier finding that the induced condition in Experiment 1 showed stronger collinearity. The results were highly similar to the results described in Table 2, with only a stronger loading of energy arousal on PC1 in the induced condition (−0.38) than in the perceived condition (0.00).

**Table 2:** PCA results for dimensional, discrete, and hybrid model showing variance explained for each PC and the loadings of each variable on those PCs.

	**Dimensional**		**Hybrid**	
	**PC 1 (64.9%)**	**PC 2 (34.8%)**	**PC 1 (71.8%)**	**PC 2 (15.7%)**
Valence	0.70	−0.20	0.41	−0.11
Tension	−0.71	−0.10	−0.37	0.38
Energy	−0.07	−0.97	0.09	0.84
	**Discrete**			
	**PC 1 (79.9%)**	**PC 2 (12.3%)**		
Anger	−0.46	0.34	−0.39	0.13
Fear	−0.43	0.39	−0.36	0.00
Sadness	−0.38	−0.82	−0.30	−0.33
Happiness	0.49	0.18	0.40	0.15
Tenderness	0.47	−0.16	0.39	−0.02

For the discrete affect model, we find that most scales load relatively equally onto the first component. This component may also be summarized as a form of valence, as it is differentiated by anger, fear, and (less strongly) sadness in one direction and happiness and tenderness in the other. For the second PC, sadness shows the strongest loading. Thus, the three dimensions may be reduced to a valence and energy model, and the five discrete affects may be reduced to a valence and sadness model.

**Figure 3:**
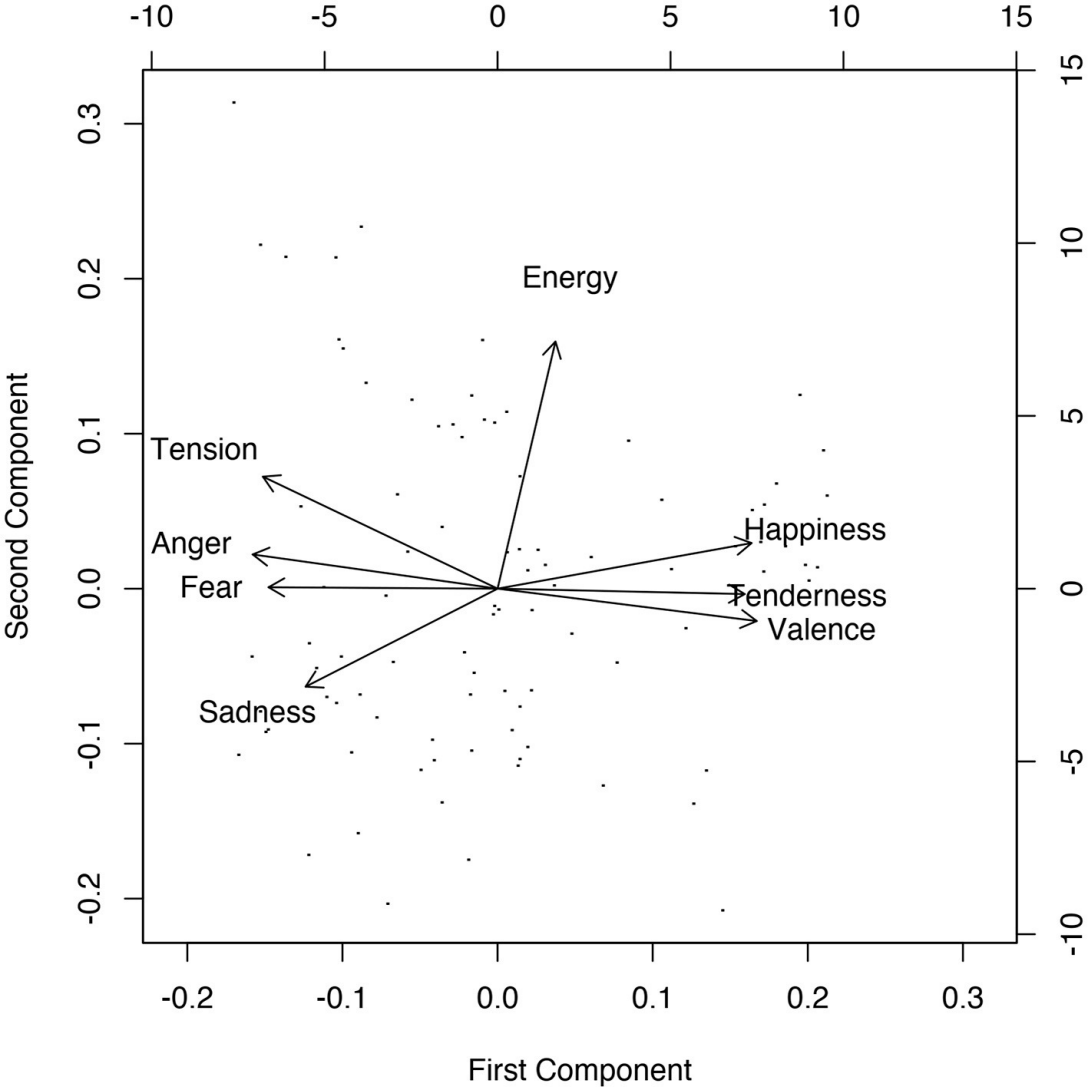
Loadings of scales and stimuli on the two rotated principal components for the hybrid PCA.

Finally, when we take all eight affect scales and run a PCA, we find two dimensions where, based on the strongest loadings, the first may be called a valence dimension and the second an energy dimension. These loadings of the hybrid PCA are also visualized in Figure 3, where we see tension arousal, anger, and fear vs. valence, happiness, and tenderness loading on the first PC and energy arousal strongly loading on the second PC. Based on the loadings of sadness, we may conclude it is a combination of the two PCs, or of valence and energy arousal.

### 3.3. Mapping

Canonical correlation analysis (CCA) tests the relationship between two sets of variables, here the dimensional and discrete affect scales, by finding linear combinations of the two sets of variables that are maximally correlated. It is another form of dimension reduction that aims to find maximum overlap. Table 3 shows the results of the CCA. All three canonical variates were found to be significant (Wilk's lambda), but the first canonical variate explained nearly all of the variance in the data, whereas the following two canonical variates explained only 1% or less. We find a similar pattern if we look at the redundancy. Redundancy represents the proportion of variance in one set of variables that is explained by the canonical variate of the other set of variables. Thus, the first canonical variate of the discrete variables explained 58% of the variance in the dimensional variables, whereas the first canonical variate of the dimensional variables explained 76% of the variation in the discrete variables. The second and third variates explained 3% or less of the variance, even though the two models did show significant canonical correlations of 0.46 and 0.32. Consequently, we focus on interpreting the first canonical variable, as it captures almost the entirety of the relationship between the dimensional and discrete affect scales. Most variables show a high loading on the first canonical variate, which may again be interpreted as a valence dimension. Sadness and especially energy show a lower loading on the first canonical variate, which indicates that they behave more independently from the other sets of variables. Thus, the two models correspond most strongly on the valence dimension, where the redundancies of the first canonical variable suggest that the dimensional scales explain more of the variance in the discrete affect scales than in the inverse case.

**Table 3:** Significance, correlation, and variation explained of canonical variates, as well as loadings and redundancy of the two affect models.

	**CV 1**	**CV 2**	**CV 3**
Wilk's lambda	*F*_(15, 229.5)_ = 39.1, *p* < 0.0001	*F*_(8, 168)_ = 3.9, *p* < 0.001	*F*_(3, 85)_ = 3.3, *p* < 0.05
Can. corr.	0.98	0.46	0.32
Var. explained	0.98	0.01	0.005
Valence	0.98	−0.09	−0.15
Tension	−0.87	0.35	0.35
Energy	0.28	0.39	0.88
Redundancy	0.58	0.02	0.03
Anger	−0.93	0.19	0.30
Fear	−0.86	0.33	−0.22
Sadness	−0.74	−0.63	−0.17
Happiness	0.97	0.12	0.19
Tenderness	0.93	−0.01	−0.17
Redundancy	0.76	0.02	0.005

**Figure 4:**
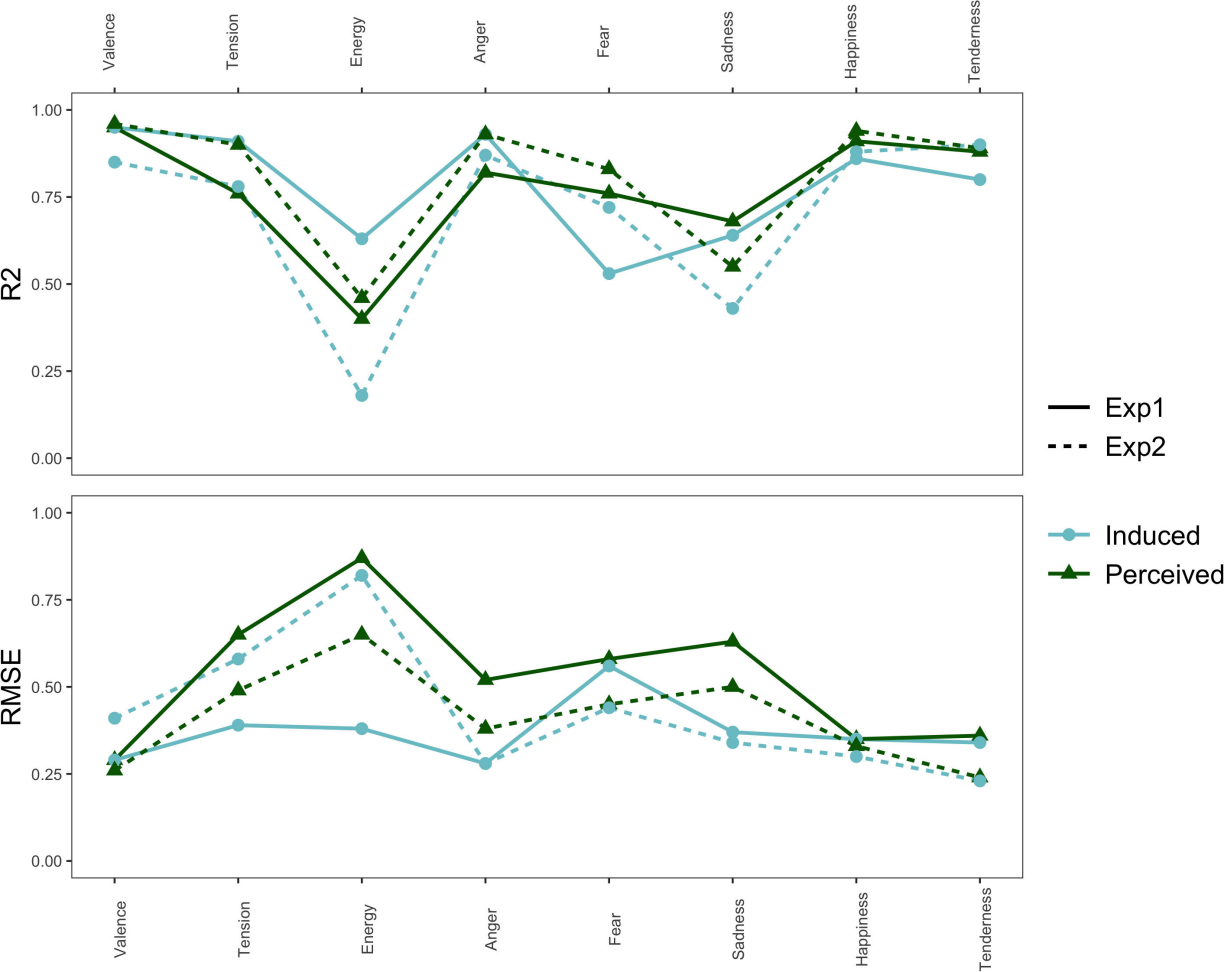
The *R*^2^ and RMSE values for each regression model predicting each of the affect scales, separated by experiment and affect locus.

We conducted linear regression analyses to further analyze how the dimensional and discrete affect scales corresponded to each other. We used the five discrete affect scales to predict each of the scales in the dimensional affect model. For example, all five discrete scales were predictors (independent variables) in the regression predicting valence (dependent variable). We also did the inverse by using the dimensional affect scales to predict each of the discrete affect scales. To measure how well the affect models correspond to each other, we used 5-fold cross-validated regression model performance. Figure 4 shows the *R*^2^ and *RMSE* values for each of the regression models. For energy arousal especially, there is a dip in *R*^2^ and a spike in *RMSE* indicating that the model predicting energy arousal performs relatively poorly, i.e., the discrete affect scales cannot fully capture or predict the dimensional scale of energy arousal. A similar, but smaller, trough and peak can be seen for the regression performance of the dimensional affect scales in predicting the discrete scale of sadness.

Table 4 summarizes the *R*^2^ and *RMSE* averaged over different groups. Comparing the average model performance of the regression models from the induced to the perceived locus (i.e., including both dimensional predicting discrete affect, and the inverse case), we see that the difference in performance is relatively small, and whereas *R*^2^ is higher for perceived affect, the *RMSE* is lower for induced affect. We see a similar small and inconsistent difference in model performance when we compare Experiments 1 and 2. However, both *R*^2^ and *RMSE* show better performance for the models in which the dimensional scales are used to predict the discrete scales than in the inverse case. Regression models that contain more predictors generally show better model performance (higher *R*^2^, lower RMSE), and consequently, the discrete scales (five predictors) predicting the dimensional scales have an advantage over the dimensional scales (three predictors) predicting the discrete scales. It is thus noteworthy that despite that, the dimensional model is actually more successful at predicting the discrete affect ratings than vice versa.

**Table 4:** Means and standard deviations for the groups summarizing affect locus, experiment, and model on *R*^2^ and RMSE.

**Variable**	**Groups**	***R^2^* (SD)**	**RMSE (SD)**
Affect locus	Induced	0.74 (0.21)	0.40 (0.15)
	Perceived	0.79 (0.18)	0.47 (0.17)
Experiment	Exp 1	0.78 (0.16)	0.45 (0.17)
	Exp 2	0.74 (0.23)	0.42 (0.16)
Model	Dis → Dim	0.73 (0.26)	0.51 (0.21)
	Dim → Dis	0.80 (0.14)	0.39 (0.11)

Dis → Dim summarizes the models where discrete affect was the independent variable and dimensional affect the dependent variable, and Dim → Dis summarizes the inverse case.

**Figure 5:**
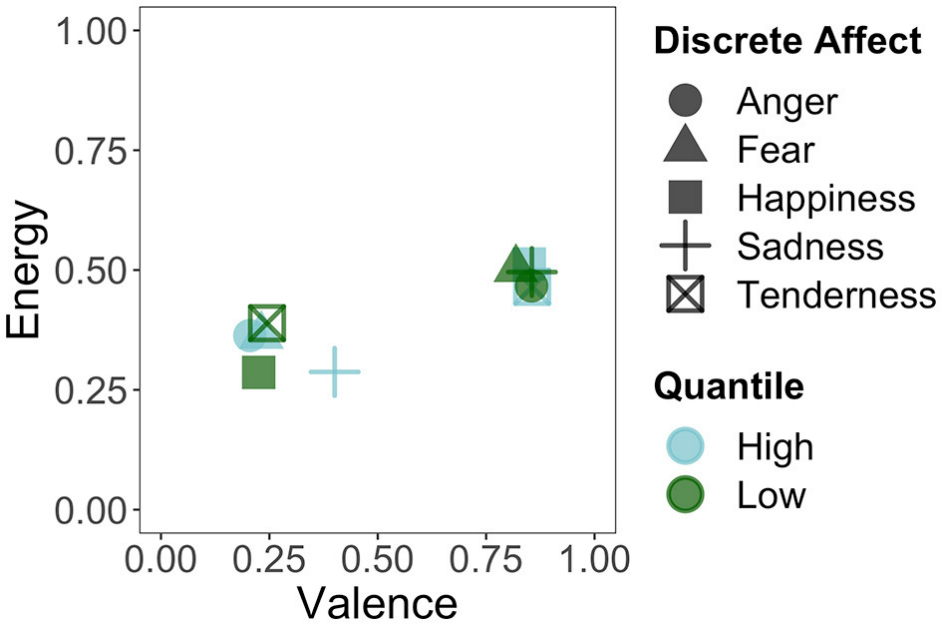
Mapping of the stimuli in the first (low) and fourth (high) quantile of the discrete affects onto the valence and energy dimensions.

To further explore the scales of energy arousal and sadness, which showed relatively poor performance in the regression models and appeared to behave more independently in the PCA, we visually mapped the discrete affects onto the dimensional affect scales and vice versa. For Figure 5, we took the stimuli that fell in the lowest (first) and highest (fourth) quantile of each of the discrete affects and calculated the mean valence and energy rating of those stimuli. Here, we can see that negative and positive valence are clearly distinguished by the discrete affects on the x axis: low happiness and tenderness, as well as high anger, fear, and sadness are mapped onto the lower, more negative end of the valence scale. High sadness, however, was not as negatively valenced as the other affects. Conversely, high happiness and tenderness, as well as low anger, fear, and sadness are mapped onto the higher, more positive end of the valence scale. For energy, however, such distinctions are much less extreme. The high and low extremes of the discrete affects all hover around the midpoint of the energy arousal scale on the *y*-axis. Thus, any variation in energy arousal that was present in the affect ratings was not captured by the discrete affect scales.

**Figure 6:**
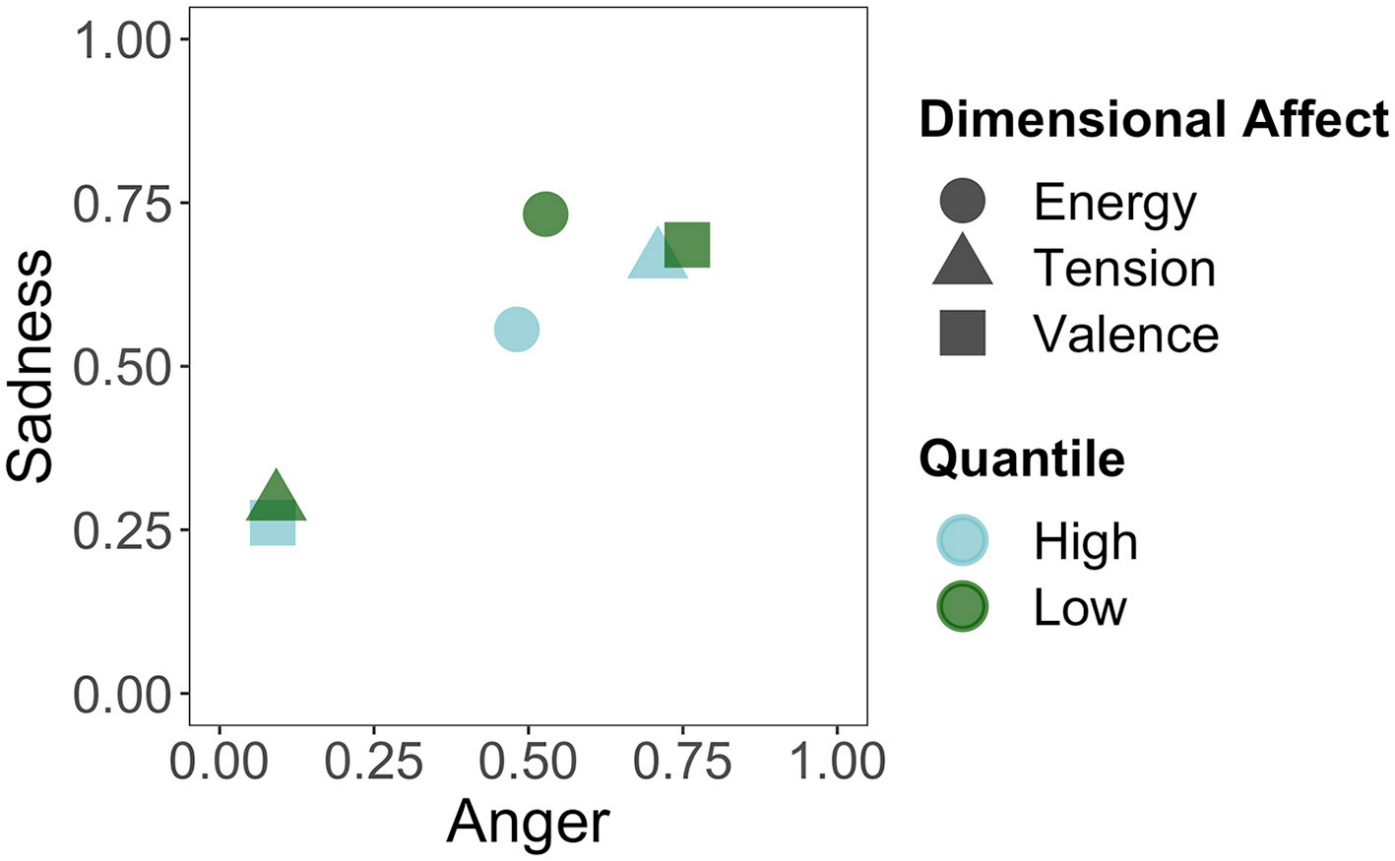
Mapping of the stimuli in the first (low) and fourth (high) quantile of the dimensional affects onto the anger and sadness categories.

Similarly, for Figure 6, we took the stimuli that fell in the lowest (first) and highest (fourth) quantile of each of the dimensional affects and calculated the mean anger and sadness ratings of those stimuli. Here, we see that the extremes of tension and valence map onto the extremes of the anger dimension. Energy hovers more around the midpoint of anger. As for sadness, we do see a greater distinction than with energy arousal in Figure 5. Lower tension and more positive valence present a lower degree of sadness, whereas higher tension and more negative valence have a higher degree of sadness. Both high and low energy map onto the higher end of sadness, although low energy is higher in sadness.

### 3.4. Individual differences

**Figure 7:**
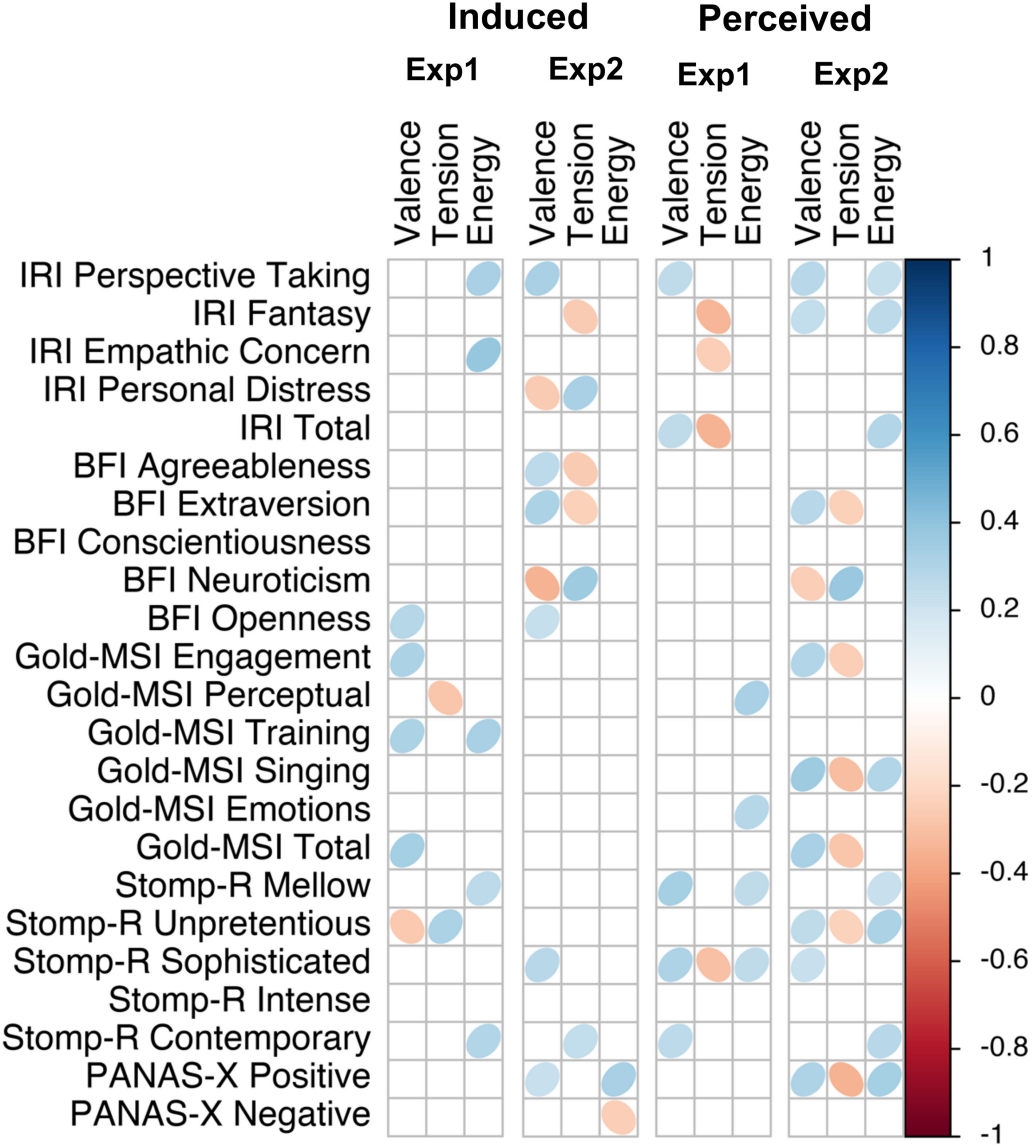
Correlation plot of questionnaire scores with dimensional affect scales, separated by experiment and affect locus.

**Figure 8:**
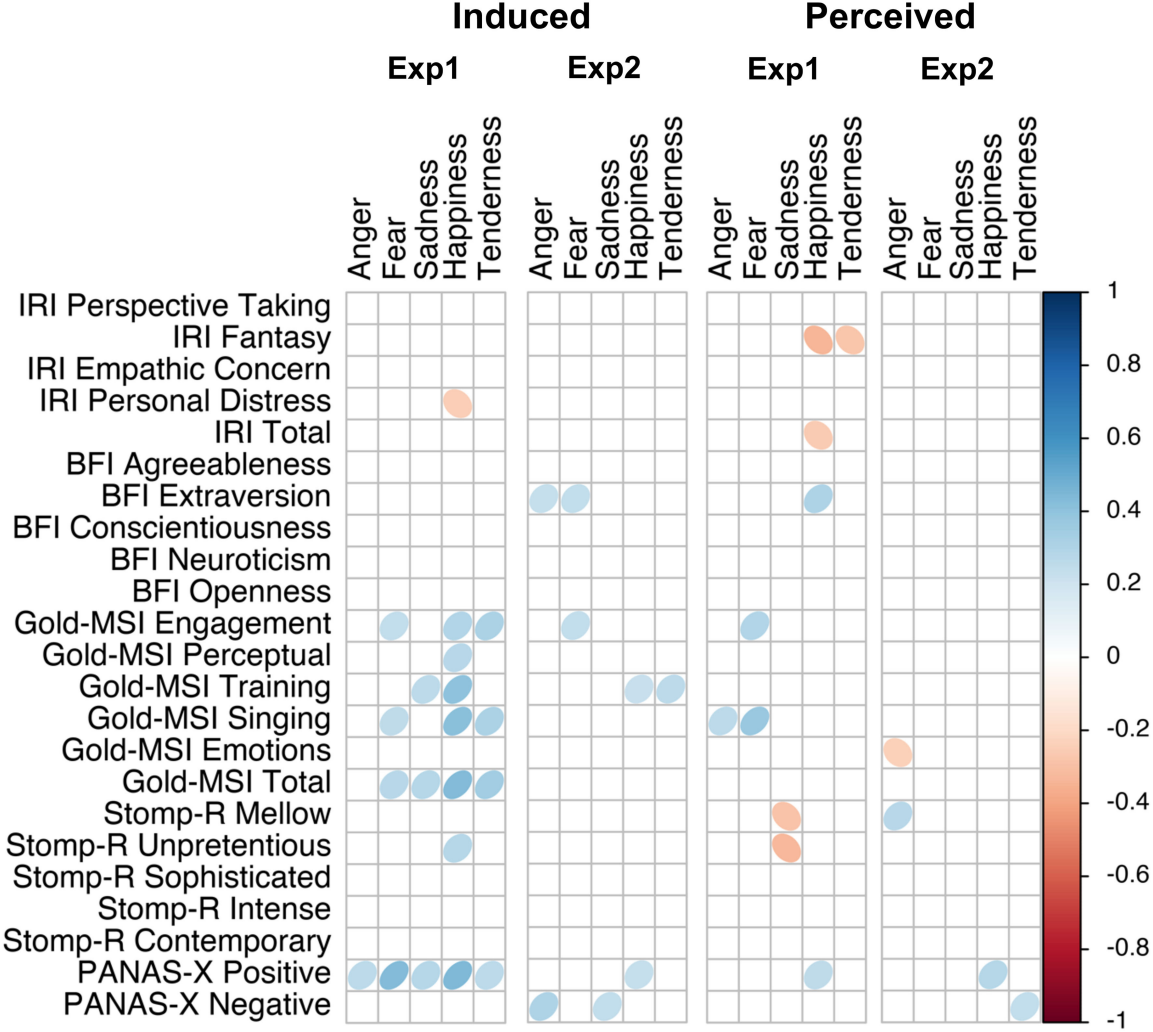
Correlation plot of questionnaire scores with discrete affect scales, separated by experiment and affect locus.

**Figure 9:**
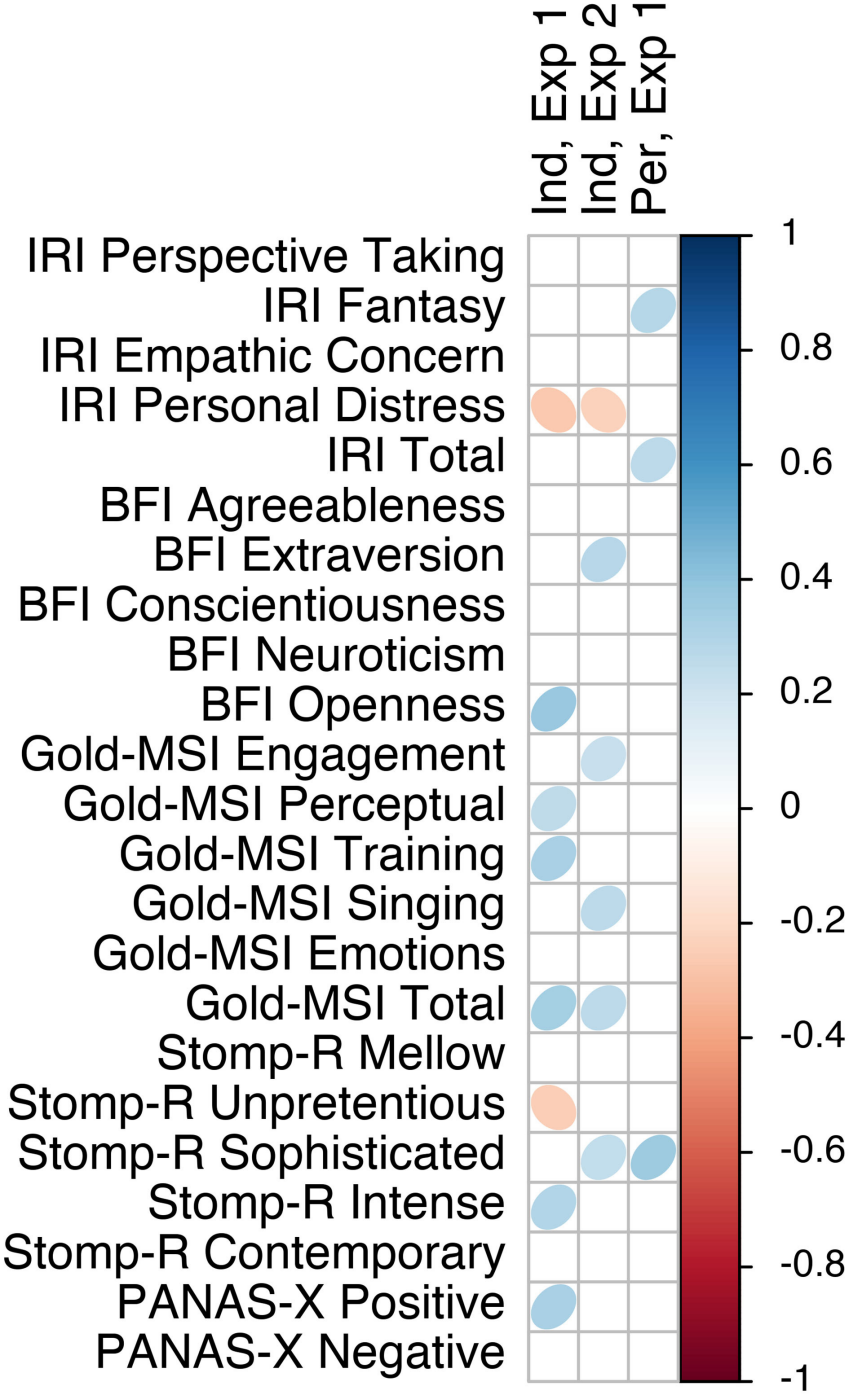
Correlation plot of questionnaire scores with preference, separated by experiment and affect locus.

We calculated Pearson correlations for each questionnaire score and sub-score with each of the affect scales, separated by experiment and affect locus. Figures 7–9 visualize the correlations of each questionnaire score with the dimensional affect, discrete affect, and preference scales, respectively. Table 5 summarizes the average absolute strength of correlations and frequency of occurrence of significant correlations of the questionnaires' (sub-)scales with the affect ratings, separated by affect locus, experiment, and affect model. The first thing we note is that none of the correlations were particularly strong, ranging from *|r|* = 0.23 to *|r|* = 0.44. When we consider the frequency of significant correlations, we find that of all the questionnaire sub-scores, pre-existing positive mood (PANAS-X positive affect) was most frequently correlated with the affect scales. This was especially noticeable in the induced ratings of discrete affect in Experiment 1: positive mood was positively correlated with anger, fear, sadness, happiness, and tenderness. Note that the direction of correlation does not change, but rather positive mood was related to increased affect ratings overall. When we average the significant correlation frequencies of the subscales of each questionnaire together, we again find that pre-existing mood was the most prevalent [PANAS-X; mean(*f*) = 9], followed by musical sophistication [Gold-MSI; mean(*f* ) = 6.7], empathy [IRI; mean(*f*) = 6], musical preferences [STOMP-R; mean(*f*) = 5.2], and Big-Five personality [BFI; mean(*f*) = 3.4]. When we consider each of the affect scales, we find that averaged over affect locus and experiment, valence was most frequently correlated with the questionnaires [mean(*f*) = 7]. There isn't one questionnaire (sub-score) that specifically stands out with respect to valence. Rather, depending on affect locus and experiment, empathy, personality, musical sophistication, preferences, and pre-existing mood all correlated with valence in one way or another.

**Table 5:** Average absolute frequency of significant correlations and correlation strength between questionnaires and affect ratings separated by affect locus, experiment, and affect model.

**Variable**	**Groups**	***f***	***|r| (SD)***
Affect locus	Induced	3.9	0.29 (0.05)
	Perceived	3.2	0.29 (0.04)
Experiment	Exp 1	3.7	0.30 (0.05)
	Exp 2	3.5	0.27 (0.04)
Model	Dimensional	5.5	0.29 (0.04)
	Discrete	2.1	0.30 (0.06)

Model groups exclude the preference ratings.

Taking the frequency and strength of correlations together, it appears that the experiment with single notes is especially susceptible to the influence of individual differences. Comparing the two affect models, the dimensional model showed more frequent correlations than the discrete model, and thus also appeared to be more susceptible to the influence of individual differences. We find that nearly all questionnaires were correlated with some affect scales, most frequently the pre-existing positive mood. A few more patterns are detectable in Figures 7, 8. For example, like PANAS-X Positive, several Gold-MSI scores also positively correlated with the discrete affect scales for the induced affect of Experiment 1, suggesting that increased musical sophistication also leads to increased intensity of induced affect. Note that we inverted the scores for tension; whereas a participants' higher rating reflected increased relaxation in the experimental procedure, for analysis we inverted the scores so that a higher rating reflected increased tension. Thus, the negative correlations with tension could be inverted to reflect relaxation, consequently rendering nearly all correlations positive. That is, a higher score on most of the questionnaire (sub-)scores is associated with higher ratings of positive valence, relaxation, energy, anger, fear, sadness, happiness, and tenderness, perhaps indicating a form of affective reactivity regardless of affective content.

Finally, we investigated the preference scale independently, as it is separate from our main focus on the dimensional and discrete affect models. First, we found that after valence, the preference ratings correlated most frequently with the questionnaire (sub-)scores, especially when participants were rating their induced affect. Figure 9 shows all the correlations with the preference scale. Increased scores of IRI Personal Distress were related to a decreased preference for the musical stimuli when rating induced affect, although IRI Fantasy and IRI Total when rating perceived affect were related to increased preference for the musical stimuli. That is, participants with the tendency to feel anxious and uneasy during tense interpersonal settings (i.e., Personal Distress) were less likely to enjoy the musical stimuli in both experiments, whereas participants with the tendency to transpose themselves imaginatively into the feelings and actions of fictitious others, and who are generally more empathetic, were more likely to enjoy the musical stimuli in the first experiment. Personality traits of extraversion and openness were also related to increased preference in the induced condition. Several Gold-MSI scores were positively correlated with preference for the stimuli in the induced condition, as well as PANAS-X Positive. Considering musical preferences (STOMP-R), preference for sophisticated music (jazz, classical, opera) and intense music (rock, punk, metal) were related to increased preference for our stimuli, but preference for unpretentious music (pop, country, religious) was related to a decreased preference.

## 4. Discussion

Based on the internal consistency and inter-rater reliability, we found that sadness scored the lowest and potentially was the most susceptible to individual differences compared to the other dimensional and discrete affect scales. Overall, there was higher consistency and agreement on perceived than on induced affect, on single notes than on chromatic scales, and on dimensional than on discrete affect scales, although these differences are minor. Zentner et al. (2008) and Vuoskoski and Eerola (2011a) similarly found higher consistency and agreement for the dimensional than for the discrete affect model.

Correlation analyses between affect scales indicated that dimension reduction in both the dimensional and discrete affect models was warranted. For the dimensional model, valence and tension were highly correlated, and dimension reduction showed that two dimensions representing valence/tension arousal on the one hand and energy arousal on the other explained most of the variation in the ratings. The inclusion of tension arousal as a third affect dimension has been a point of contention. Whereas, some studies also find that valence and tension arousal are highly collinear (Krumhansl, 1997; Eerola and Vuoskoski, 2011; Vuoskoski and Eerola, 2011a; Eerola et al., 2012), interestingly, the study with stimuli and experimental design closest to our first experiment did not find high collinearity between these two affect dimensions [McAdams et al., 2017; *r*(135) = 0.46]. The key difference between the current experiments and the one by McAdams and colleagues are the participant pool and testing environment, i.e., university-based vs. world-wide and in-lab vs. online. Our finding that there was either no significant or only a weak correlation between energy and tension arousal is, however, more similar to McAdams et al.'s findings than those of Eerola et al. (2012). McAdams et al. explain this incongruity by the lack of pitch variation in Eerola et al.'s (2012) experiment, which was present in McAdams et al.'s and the current experiments, where energy arousal appears to be mostly related to spectral variability due to changes in pitch. Furthermore, although Eerola et al. (2012) did find a strong correlation between valence and preference, as did the current study, McAdams et al. (2017) did not. Preference may be considered a fundamental manifestation of affect (Zajonc, 1980) and has been previously used as a substitute measure for valence, alongside arousal (Brown et al., 2004). The similarities and differences we find in our results further argue for the systematic comparison of affect models in different experimental contexts, as there may not be a one-size-fits-all model appropriate for representing perceived and induced affect in music.

For the discrete affect model, anger, fear, happiness, and tenderness all correlated strongly with each other, and sadness did so to a lesser extent. Similarly, a PCA showed that two components representing anger/fear/happiness/tension and sadness explained most of the variation in the ratings. Although Eerola and Vuoskoski (2011) also found that most of the discrete ratings of perceived affect correlated with each other, the correlations were not as strong as in the present study. Nevertheless, they did find that most of the discrete affect categories could be mapped onto a single valence dimension, and sadness onto a second dimension. Similarly, in their study on induced affect, Vuoskoski and Eerola (2011a) found that most of the variance in the discrete affect model could be represented by two components of valence (or tension) and energy. Furthermore, when putting all affect scales together in the current experiment, again two components explained most of the variation in ratings, with the highest loading for valence on the first component, and energy arousal on the second component. Based on the correlations and PCAs, we conclude that, be they dimensional or discrete, two components captured most of the variance in induced and perceived affect related to the timbres of single notes and chromatic scales.

Canonical correlations and predictive modeling showed that the correspondence of the two affect models is relatively high, in particular on the valence dimension. The regression modeling performance overall was higher for perceived than induced affect, especially in response to single notes. Thus, when assessing perceived affect with short stimuli varying in timbre, the dimensional and discrete affect models appear to be mostly capturing the same affective responses and may thus be considered interchangeable. Furthermore, regression model performance and canonical redundancy indicate that a dimensional model of affect captures more variance in the discrete affect ratings than vice versa. This result suggests that the dimensional affect model is more appropriate for capturing the affective response to music, which concurs with previous dimensional and discrete comparisons in perceived and induced affect (Eerola and Vuoskoski, 2011; Vuoskoski and Eerola, 2011a). Further investigation of energy arousal revealed that it varied in a manner that was not captured by any of the discrete affect scales. Although the same was true for sadness, valence and tension still appeared to capture some of the variation that was present in the sadness ratings, whereas both low and high energy were associated with increased sadness.

Recent studies suggest that there are different kinds of musical sadness that may be distinguished by their energetic level (Warrenburg, 2020): melancholy (low energy) vs. grief (high energy). In the current study, sadness also appeared to load on both principal components, as perhaps a combination of valence and energy. Additionally, Eerola and Vuoskoski (2011) found that sadness did not correlate with valence, and although these two scales did correlate in the present study, the sadness scale showed the weakest correlations of all the discrete scales overall. Sadness also correlated negatively with preference, but this was weaker than the correlation of preference with other unpleasant affects such as tension, anger, and fear. We were unable to directly test the role of individual differences in the preference for sadness, a topic of particular interest in musical affect research (Eerola et al., 2018), as sadness and preference were rated by different groups of participants. Our results and previous studies suggest that sadness is not necessarily associated with negative valence and behaves more independently from the other discrete affect measures (Bigand et al., 2005; Kreutz et al., 2008).

Finally, our exploration of the role of individual differences showed that all of the measures correlated with affect ratings in one way or another, with a moderate correlation strength at most. Of all the affect scales, valence was significantly correlated the most frequently with the measures, indicating that it was the most susceptible to be influenced by individual differences. Comparing the dimensional and discrete models, on average, dimensional affect scales correlated more frequently with individual difference measures, also indicating they are more susceptible to influence by these differences. Similarly, the first experiment showed more frequent and stronger correlations than the second experiment, suggesting that a short exposure time allowed for more individual variability. After valence, preference was most influenced by individual differences from all included sources (pre-existing mood, dispositional empathy, personality, musical sophistication, and musical preferences). This contrasts with our findings on internal consistency and inter-rater reliability, as discussed above, which suggested that the discrete model, the second experiment, and specifically the scale of sadness would be more susceptible to individual differences. The differences in consistency and agreement perhaps do not reflect the individual differences we measured but may be caused by other sources of variation.

Pre-existing positive mood most frequently influenced the affect ratings. Interestingly, this influence was unidirectional; a positive mood led to higher ratings on most scales, suggesting a higher emotional reactivity, or intensity, overall. Previous research has also shown that extraversion, related to experiencing more positive affects, and pre-existing mood were related to affective processing or intensity (Vuoskoski and Eerola, 2011a,b). Following positive mood, several musical sophistication scores were also frequently correlated with the affect ratings, mostly unidirectionally. Whereas, some studies found little or no difference in affective response between musicians and non-musicians (David Frego, 1999; Bigand et al., 2005; Filipic et al., 2010), other studies did (Egermann and McAdams, 2013; McAdams et al., 2017). Part of this discrepancy may be a result of the ambiguous definition of musicianship. The broader and more inclusive use of musical sophistication in the current study does corroborate the importance of musical expertise in the musical affective response to music, although musical sophistication may not be directly related to the degree of formal musical training. After pre-existing mood and musical sophistication, we found that dispositional empathy, musical preferences, and Big-Five personality were most frequently correlated with the affect scales. The manner in which individual differences correlated with affect scales varied between scales, experiments, and affect loci, which makes it difficult to hypothesize about the underlying mechanisms connecting individual differences and affective response. This does again indicate, however, that the experimental context can influence one's findings. Furthermore, these results show that many sources of individual differences may influence the affective response to music, although it should be noted that with such a large number of comparisons, a few of the significant correlations are likely to be spurious.

To summarize, the aim of this study was to compare the performance of the discrete and dimensional affect models, measuring perceived and induced affect, in response to relatively short, affectively ambiguous musical sounds, as tested in an online environment. Both affect models' scales could be reduced to two components. Furthermore, whereas the dimensional and discrete affect models appeared to be relatively interchangeable, energy arousal varied in a manner that was not captured or predicted by the discrete affect model. Future research may further explore the role of energy compared to discrete affects (Warrenburg, 2020) and how it relates to changes in pitch height (McAdams et al., 2017). Finally, our exploration of individual differences showed that pre-existing mood, dispositional empathy, Big-Five personality, musical sophistication, and musical preferences all played a role in the affective response to music. Future studies may thus either consider these factors in their investigation of the mechanisms underlying musical affect or ensure their participant population is appropriately represented on those factors to ensure generalizability.

## Data availability statement

The original contributions presented in the study are publicly available. This stimuli, data, and R scripts can be found here: https://doi.org/10.5683/SP3/2BZJRF.

## Ethics statement

The studies involving humans were approved by McGill University Research Ethics Board II. The studies were conducted in accordance with the local legislation and institutional requirements. The participants provided their written informed consent to participate in this study.

## Author contributions

IK: Conceptualization, Data curation, Formal analysis, Investigation, Methodology, Visualization, Writing—original draft. MM: Data curation, Formal analysis, Methodology, Writing—review & editing. AW-M: Writing—review & editing. SM: Supervision, Writing—review & editing.
